# Different acupuncture therapies for spastic paralysis after stroke: A protocol for systematic review and network meta-analysis

**DOI:** 10.1097/MD.0000000000020974

**Published:** 2020-07-02

**Authors:** Ruiqi Wang, Rongfang Xie, Jinwen Hu, Qingzhong Wu, Wangfu Rao, Chunhua Huang

**Affiliations:** a Jiangxi University of Traditional Chinese Medicine; b Affiliated Hospital of Jiangxi University of Traditional Chinese Medicine, Nanchang, Jiangxi Province, China.

**Keywords:** acupuncture, network meta-analysis, protocol, spastic paralysis after stroke

## Abstract

**Background:**

Stroke is emerging as a significant health issue that threatens human health worldwide and as a common sequela of stroke spastic paralysis after stroke (SPAS) has received wide attention. Currently, several systematic reviews have suggested that the commonly used acupuncture therapy (electroacupuncture, fire acupuncture, warm acupuncture, and filiform needle acupuncture) has achieved significant efficacy in the treatment of SPAS. In this study, network meta-analysis will be used to analyze the results of different clinical trials and evaluate the differences in the efficacy of different acupuncture treatments for SPAS.

**Methods:**

Only randomized controlled trials will be included and all patients were diagnosed as spastic paralysis after stroke. A computer-based retrieval will be conducted at CNKI, WanFang databases, VIP, Sinoed, Pubmed, Embase, Web of Science, and the Cochrane library. The search period limit is from the time the date of database establishment to April 17, 2020. To avoid omissions, we will manually retrieve relevant references and conference papers. The risk of bias in the final included studies will be evaluated based on the guidelines of the Cochrane Handbook for Systematic Reviews of Interventions. All data analysis will be conducted by Revman5.3, WinBUGS 1.4.3, and Stata14.2.

**Results:**

This study quantified the effectiveness of each intervention for different outcome indicators. The primary outcomes include the Fugl-Meyer Assessment score, the modified Ashworth scale for the assessment of spasticity, and Barthel Index. The secondary outcomes include clinical effectiveness and adverse reactions.

**Conclusion:**

It will provide evidence-based medical evidence for clinicians to choose more effective acupuncture therapy for SPAS

## 1 Introduction

Stroke is emerging as a significant health issue that threatens human health worldwide. Besides a previous study shows stroke is a leading cause of death in China.^[1,2]^ As 1 of the most common sequelae and a major obstacle for patients to recover, the incidence rate of spastic paralysis after stroke (SPAS)is arising in about 30% of patients.^[3,4]^ However, the rate of spasms is highly variable and can occur in the short, medium, or long term Post-stroke period.^[5]^ Relevant articles revealed the possible etiologies that mainly injured the upper motor neurons, resulting in a significant increase in muscle tension.^[6,7]^ If failure to take active treatment may cause Joint deformation, muscular atrophy, and pain, which may affect the rehabilitation of the limbs and the quality of life.^[8]^

Nowadays, the common treatment methods for SPAS are medications, surgical interventions, and physical therapy.^[9,10]^ However, concerns have been raised about the side effects of the drugs, the invasive nature of the surgery, and the high cost of treatment. Due to limitations of current SPAS treatment, it is particularly important to explore alternative treatment options with better efficacy, fewer side effects, and low price. As a part of traditional Chinese medicine treatment, acupuncture has been used to treat stroke patients for many years in China and become more popular in Western countries in recent years.^[11]^ Modern research shows that acupuncture can enhance immunity, regulate blood circulation, relieve the pain, help muscles relax and exercise more passively, thereby increasing recovery.^[12–14]^

More and more systematic reviews suggest that acupuncture plays an important role in patients with SPAS.^[15–19]^ But traditional systematic reviews can only directly compare 2 interventions, and cannot compare multiple acupuncture therapies. At present, there are various types of acupuncture, the 4 acupuncture therapies (electroacupuncture [EA], fire acupuncture (FA), warm acupuncture (WA), filiform needle acupuncture) analyzed in this study are commonly used methods. Meanwhile the advantages of various acupuncture therapies are not the same, so the choice of which acupuncture therapy can bring greater curative effect has brought troubles to clinical operators. In this study, the effectiveness of acupuncture therapy commonly used in clinical will be ranked by network meta-analysis, to provide scientific evidence-based medicine basis for the clinical selection of the best.

## 2 Protocol registration

This systematic review protocol will be reported strictly adherence to the Preferred Reporting Items for Systematic Review and Meta-analysis Protocols (PRISMA-P).^[20]^ The protocol of the systematic review has been registered in the INPLASY website(registration number is INPLASY202050058). If there are any adjustments during the entire study period, we will fix and update the detailed information in the final report in time.

## 3 Methods

### 3.1 Inclusion criteria

#### 3.1.1 Study type

Only randomized controlled trials (RCTs) will be included and no restrictions on language. Non-RCTs such as system reviews, meeting abstracts will be excluded. Interventions incompatible with the inclusion criteria and animal experiments will be excluded. Duplicate publications are preferred to the 1 with the most recent and comprehensive data.

#### 3.1.2 Participants

All adult patients are diagnosed as SPAS. There will also be no restrictions based on gender, race, and the course of the disease. However, the following patients will be excluded:

(1) Patients with mental illness who cannot cooperate with treatment.
(2) Patients with complications or vital organ failure.
(3) Pregnant or lactating women.
(4) Patients with adverse reactions or pain that cannot withstand acupuncture.

#### 3.1.3 Interventions

The experimental group only use EA, FA, WA or milli-acupuncture, and the control group uses rehabilitation or a comparison of the above 4 acupuncture methods. Patients in both groups could receive conventional medical treatment and treatment duration and frequency are not limited.

#### 3.1.4 Outcome indicators

The primary outcomes include the Fugl-Meyer Assessment score (FMA), the modified Ashworth scale (MAS) for the assessment of spasticity, and Barthel Index (BI). These indexes can evaluate the recovery of limb spasm.^[21–23]^ The secondary outcomes include clinical effectiveness and adverse reactions. Clinical effectiveness including 4 grades of recovery, obvious effect, effective and ineffective. Effective rate = ([recovery + obvious effect + effective]/total number of cases) × 100%.

### 3.2 Data sources and search strategies

A computer-based retrieval will be conducted at CNKI, WanFang databases, VIP, SinoMed, Pubmed, Embase, Web of Science, and the Cochrane library. The search period limit is from the time the date of database establishment to April 17, 2020. To ensure the comprehensiveness of the search, relevant references and conference literature are also included. A search strategy is as follows:(((((((([stroke [MeSH Terms]) OR (cerebral infarction[MeSH Terms])) OR (cerebral hemorrhage[MeSH Terms])) OR (sequelae)) OR (spasm)) OR (spastic paralysis)) OR (spastic hemiplegia)) AND ((((((((acupuncture) OR (EA)) OR (FA)) OR (WA)) OR (filiform needle acupuncture)) OR (warm needle)) OR (fire-needle)) OR (needle acupuncture))) AND ((((“Randomized Controlled Trial” [Publication Type]) OR “RCT” [Publication Type]) OR “clinical trials” [Publication Type])).

### 3.3 Selection of studies and data extraction

Two evaluators (RQW and RFX) will independently screen all relevant literature according to the inclusion and exclusion criteria. Then import them into Endnote for centralized management. We then go through the title abstract and the full text, in turn, to determine which study ultimately meets the criteria. During this process, if there is any dispute, the decision will be made by the third evaluator (JWH). Also, we will establish a document information extraction table in EXCEL, the extracted data includes the following: title, author, publication time, abstract, sample size, number of cases in each group, age, gender, course of the disease, distribution method, intervention, treatment course, outcome indicators, and so on.

### 3.4 Risk assessment of bias

The risk of bias in the final included studies will be evaluated based on the guidelines of the Cochrane Handbook for Systematic Reviews of Interventions. The evaluation criteria include 7 items: selections bias, performance bias, detect bias, attrition bias, reporting bias, and other bias. Each item will be graded into 3 levels: “high risk”, “low risk”, and “not clear”.^[24,25]^ This work will also be done independently by 2 reviewers.

### 3.5 Statistical analysis

Revman 5.3 software is used for bias evaluation and traditional Meta-analysis. For continuous variables (FMA, MAS, BI), the results will be reported as mean difference (MD) with 95% confidence interval (CI); Count data (clinical effectiveness and adverse events) will be calculated with the odds ratio (OR) and 95% CI. WinBUGS 1.4.3 and Stata 14.2 are used for network meta-analysis.^[26,27]^ In the WinBUGS software, Bayesian network meta-analysis is performed by the Markov Chain Monte Carlo (MCMC) method, which is simulated by 4 chains, the number of iterations is set to 50,000, and the step size is set to 10.^[28]^ At the same time, the potential scale reduction factor (PSRF) is used to evaluate the convergence of the results. When 1.00≤PRSF≤1.05, it indicates that the results converge well and the results obtained are highly reliable.^[29]^

We can calculate the surface under the cumulative ranking curve (SUCRA) to estimate the possible ranking order of various interventions.^[30]^ The SUCRA value ranges from 0 to 100. The larger the value, the intervention is considered to have better efficacy.

### 3.6 Assessment of inconsistency

Because of the large number of interventions involved in this study, the Loop inconsistency test of studies with direct and indirect evidence is needed in the evidence network for each outcome indicator. Calculate the inconsistency factor (IF), and judge whether there is inconsistency according to the size of IF value and *P*-value.^[30]^ At the same time, the node split model will be used to judge whether each node has local inconsistency.^[31]^ If *P* > .05, there is no significant inconsistency, and the consistency model is used. As for the results obtained from the consistency model analysis, the stability of the results can be tested by the inconsistent model .^[32,33]^

### 3.7 Heterogeneity, subgroup analysis, and sensitivity analysis

The heterogeneity between trials is quantified with the *I*^2^ and *P* values.^[34]^ For the test results with obvious heterogeneity, the source of heterogeneity should be analyzed. Subgroup analysis can be conducted according to the different sources of heterogeneity, such as the following aspects: treatment duration, disease course, underlying disease, race, gender, age, and so on. If no clear source of heterogeneity can be found, only descriptive analysis can be conducted.

The purpose of sensitivity analysis is to eliminate low-quality studies and different statistical models.^[35]^ Then, the strength, reliability, and stability of the results will be analyzed by observing the heterogeneity of different tests and whether the combined results changed after various treatments.

### 3.8 Assessment of publication bias

If the outcome indicators include in study ≥10, funnel plots will be used to assess the publication bias of the included trials.^[36]^ If there is a difference in symmetry or distribution, there will be a publication bias or a small sample effect.

### 3.9 Ethics and dissemination

Due to this is a protocol for systematic review and network meta-analysis, all data of this study are from published studies and do not involve patients, so ethical approval will not be necessary. The findings of this study will be disseminated to a peer-reviewed journal and presented at a relevant conference.

## 4 Discussion

With the continuous development and improvement of acupuncture technology, there are a variety of acupuncture treatments that are considered to be effective in the treatment of SPAS. However, the therapeutic advantages of different acupuncture therapies are different from each other, which confuses clinical choices. Network meta-analysis overcomes the shortcomings of traditional systematic reviews and can integrate direct and indirect evidence. Besides, it provides an intuitive comparison of the efficacy and safety of available technologies. So the results can provide clinicians and patients with the ability to determine the best treatment options. We will use the network meta-analysis method to assess the effectiveness of 4 commonly used clinical acupuncture manipulations, and quantified their effectiveness according to different outcome indicators.

## Author contributions

**Conceptualization:** Ruiqi Wang, Chunhua Huang, Rongfang Xie

**Data curation:** Ruiqi Wang, Chunhua Huang, Jinwen Hu

**Formal analysis:** Ruiqi Wang, Chunhua Huang, Rongfang Xie

**Project administration:** Ruiqi Wang, Chunhua Huang, Qingzhong Wu

**Supervision:** Ruiqi Wang, Chunhua Huang, Wangfu Rao

**Writing – review & editing:** Ruiqi Wang, Chunhua Huang, Rongfang Xie
